# Disparate Patient Advocacy When Facing Unaffordable and Problematic Medical Bills

**DOI:** 10.1001/jamahealthforum.2024.2744

**Published:** 2024-08-30

**Authors:** Erin L. Duffy, Melissa A. Frasco, Erin Trish

**Affiliations:** 1 Leonard D. Schaeffer Center for Health Policy & Economics, University of Southern California, Los Angeles; 2 Alfred E. Mann School of Pharmacy and Pharmaceutical Sciences, University of Southern California, Los Angeles

## Abstract

**Importance:**

People in the US face high out-of-pocket medical expenses, yielding financial strain and debt.

**Objective:**

To understand how households respond to medical bills they disagree with or cannot afford.

**Design, Setting, and Participants:**

A retrospective cohort study was carried out using a survey fielded between August 14 and October 14, 2023. The study included a random sample of adult (aged ≥18 years) survey respondents from the Understanding America Study (UAS). Participant responses were weighted to be nationally representative. The analysis took place from November 3, 2023, through January 8, 2024.

**Main Outcomes and Measures:**

Respondents reported if their household received a medical bill that they could not afford or did not agree with in the prior 12 months, and if anyone contacted the billing office regarding their concerns. Those who did reach out were asked about their experience and those who did not were asked why.

**Results:**

The survey was sent to 1233 UAS panelists, of which 1135 completed the survey, a 92.1% cooperation rate. Overall, 1 in 5 of the 1135 respondents received a medical bill that they disagreed with or could not afford. Leading bill sources were physician offices (66 [34.6%]), emergency room or urgent care (22 [19.9%]), and hospitals (31 [15.3%]), and 136 respondents (61.5%) contacted the billing office to address their concern. A more extroverted and less agreeable personality increased likelihood of reaching out. Respondents without a college degree, lower financial literacy, and the uninsured were less likely to contact a billing office. Among those who did not reach out, 55 (86.1%) reported that they did not think it would make a difference. Of those who reached out, 37 (25.7%) achieved bill corrections, better understanding (16 [18.2%]), payment plans (18 [15.5%]), price drop (17 [15.2%]), financial assistance (10 [8.1%]), and/or bill cancellation (6 [7.3%]), while 32 (21.8%) said that the issue was unresolved and 23.8% reported no change. These outcomes aligned well with respondents’ billing concerns with financial relief for 75.8% of respondents reaching out about an unaffordable bill, bill corrections for 73.7% of those who thought there was mistake, and a price drop for 61.8% of those who negotiated.

**Conclusions and Relevance:**

This cross-sectional survey of a representative sample of patients in the US found that most respondents who self-advocated achieved bill corrections and payment relief. Differences in self-advocacy may be exacerbating socioeconomic inequalities in medical debt burden, as those with less education, lower financial literacy, and the uninsured were less likely to self-advocate. Policies that streamline the administrative burden or shift it from patients to the billing clinician may counter these disparities.

## Key Points

**Question:**

How do households respond to medical bills they disagree with or cannot afford?

**Findings:**

In this cross-sectional survey of a representative sample of people in the US, 1-in-5 reported receiving a medical bill they disagree with or cannot afford; 61% of them reached out to the billing office to address their concern; less educated respondents, those with lower financial literacy, and the uninsured were less likely to reach out to a billing office. Most respondents who reached out reported financial relief, bill corrections, or better understanding of the bill.

**Meaning:**

Differences in self-advocacy may be exacerbating socioeconomic inequalities in medical debt burden.

## Teaser

This cross-sectional survey of a representative sample of people in the US examines how households respond to medical bills they disagree with or cannot afford.

## Introduction

More than half (57%) of US adults have been burdened by medical bills within the past 5 years, and the burden is higher among those with lower levels of education, lower income, women, and Black and Hispanic adults.^1^ Socioeconomic and demographic disparities in the financial burden of health care are shaped by insurance coverage, utilization, and ability to pay. There may also be differences in individuals’ willingness and ability to navigate avenues of financial relief and bill correction that contribute to disparate burden.

With high out-of-pocket costs, the onus is on patients to self-advocate by identifying billing errors, seeking financial assistance, and setting up payment plans. These processes can be complex, confusing, time intensive, and administratively cumbersome for patients.^2^ These challenges are widely recognized, and books, blogs, and podcasts have emerged in recent years informing people about how to navigate their medical bills.^3,4,5^ Yet, administrative barriers have been shown to yield disparate effects on enrollment in social programs and benefits from welfare promoting policies.^6,7^

Recent surveys have documented uninsurance, underinsurance, patients’ financial strain, and medical debt.^8,9,10^ These surveys describe utilization and resulting cost burden, but do not delve into the intermediate step of patients’ receiving and responding to bills they disagree with or cannot afford. To bridge this gap in knowledge about how patients navigate medical bills, a nationally representative panel was surveyed to probe experiences with medical billing.

## Methods

### Participants and Procedures

Participants in the Understanding America Study (UAS), which is maintained by the Center for Economic and Social Research (CESR) at the University of Southern California, were sampled in this study. The UAS is a probability-based internet panel of approximately 13 000 respondents residing in the United States (Supplement 1).^11^ Recruitment of the initial panel was by paper survey (41.3% response rate). Respondents answered surveys on a computer, tablet, or smartphone. The University of Southern California institutional review board approved this study and written informed consent was obtained. This report follows the American Association for Public Opinion Research reporting guidelines for survey studies. The analysis took place from November 3, 2023, through January 8, 2024.

A sample of 1233 panelists were recruited to respond to this survey between August 14 and October 14, 2023. Respondents’ sample weights were calculated using a 2-step approach to make the survey dataset representative of the US population aged 18 years and older with respect to a predefined set of sociodemographic variables. The first step computes base weights to correct for unequal probabilities of sampling UAS members. The second step generates a final poststratification weight, allowing the sample to align with the reference population along certain sociodemographic dimensions.

### Measures

Respondents were asked if their household received a medical bill that they could not afford or did not agree with in the prior 12 months. If yes, they were asked about the source of the bill and if anyone reached out to the billing office to address their concerns. Respondents who did not reach out were asked why not. Those who did reach out were asked a series of questions about their experience, including the objective for reaching out and the outcome, modality, time, and feelings associated with the interaction. The full survey instrument is shown in Supplement 1.

Information about respondents from previous UAS surveys was used in analyses, including demographics (age, gender, marital status, race and ethnicity, national origin), socioeconomic characteristics (education, household income), insurance type (private, Medicaid, Medicare, military, uninsured), number of chronic conditions, financial literacy score (score range, 0-14),^12^ and Big Five personality scores: extroversion (score range, 8-40), agreeableness (score range, 9-45), conscientiousness (score range, 9-45), neuroticism (score range, 8-40), and openness (score range, 10-50).^13,14^ Details about these measures are provided in Supplement 1.

### Statistical Analysis

**Figure 1.**
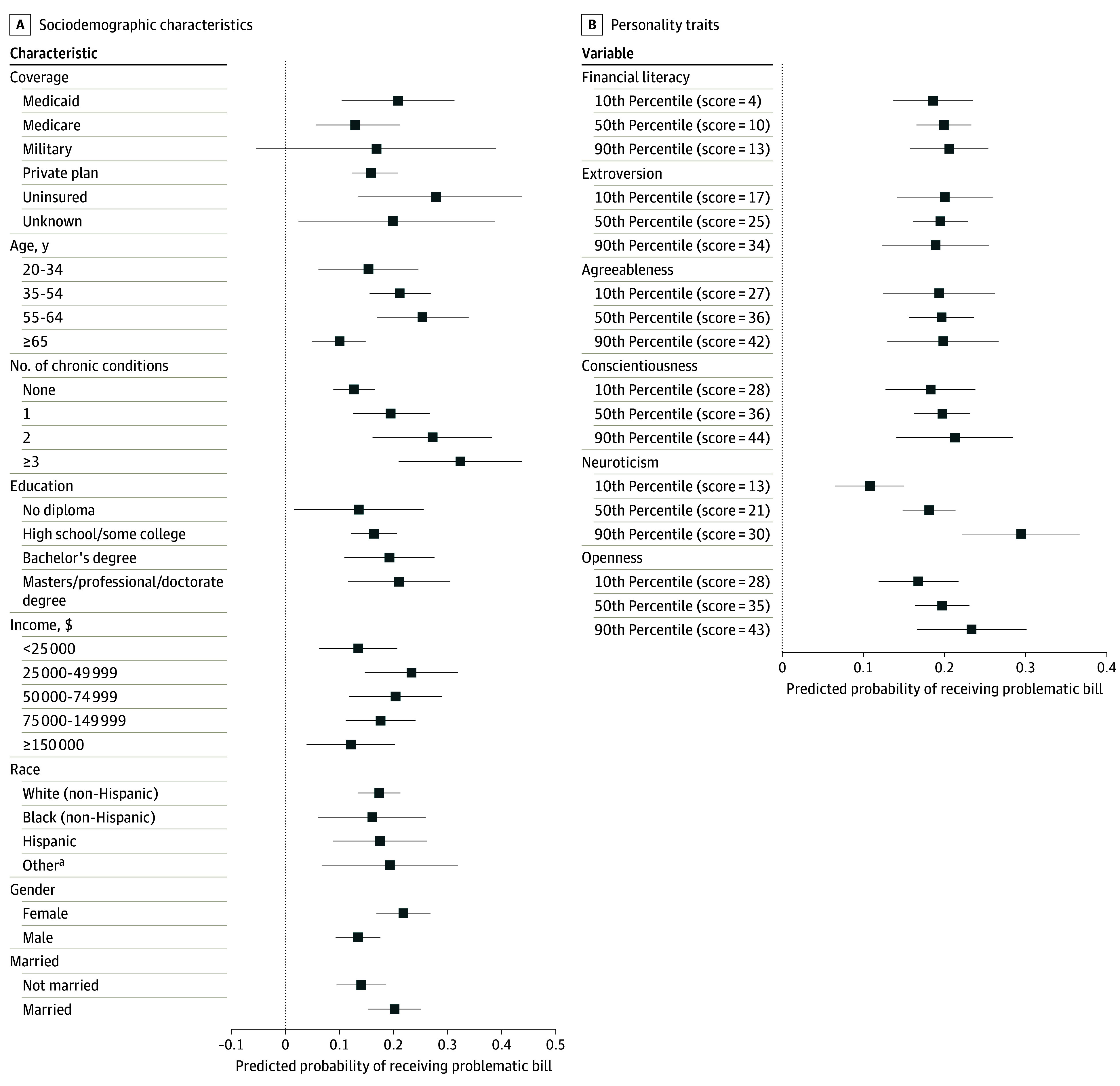
Predicted Probability of Receiving a Problematic Bill by Demographic, Socioeconomic, Financial Literacy, and Personality Characteristics Data points shown are predicted probabilities from multivariable probit regression models, with whiskers displaying the 95% CIs for these estimates. The corresponding regression models are reported in Supplement 1. ^a^Other indicates participants who did not self identify as Black, Hispanic, or White.

**Figure 2.**
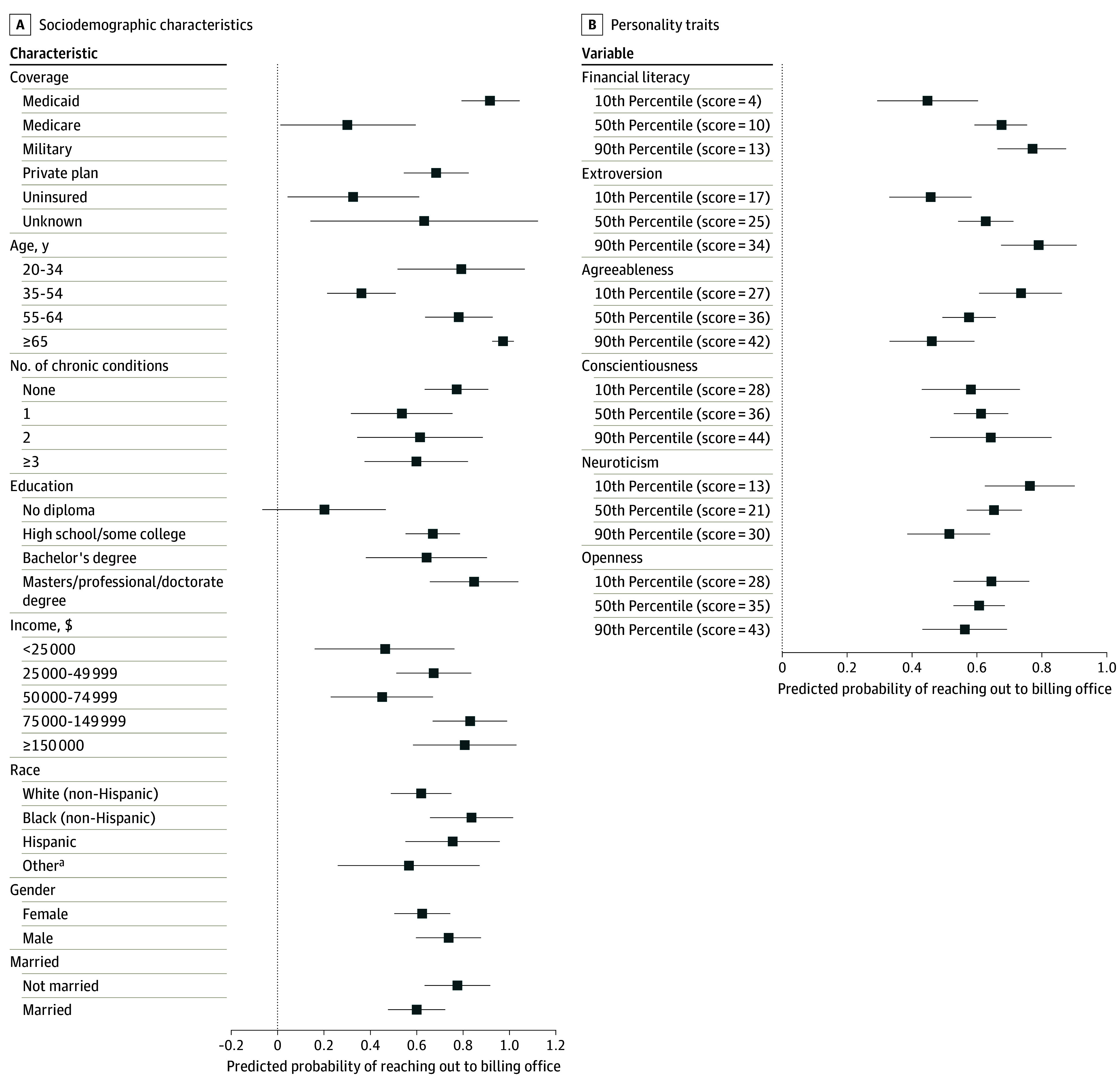
Predicted Probability of Reaching Out to the Billing Office by Demographic, Socioeconomic, Financial Literacy, and Personality Characteristics Data points shown are predicted probabilities from multivariable probit regression models, with whiskers displaying the 95% CIs for these estimates. The corresponding regression models are reported in Supplement 1. ^a^Other indicates participants who did not self identify as Black, Hispanic, or White.

Raw count values and weighted percentages with 95% CIs are reported in describing the sample characteristics and survey responses. χ^2^ tests were used to compare the attributes of respondents who did and did not reach out to the billing office, conditional on receiving a problematic bill. Weighted multivariable probit regression models were used to assess the association between respondent characteristics and the outcomes of reporting receipt of a problematic bill and, conditional on receiving a problematic bill, whether or not anyone reached out to address the concern. For each of these 2 outcomes, 1 model was fit assessing association with demographic and socioeconomic characteristics and a second model was fit assessing association with financial literacy and personality. Separating these 2 types of characteristics addresses concerns about overfitting and collinearity, and also enables discrete measurement of traits that are more easily observed and traits that require in-depth skill and personality testing. Regression results are reported in Supplement 1 and presented as predicted probabilities with 95% CIs in Figure 1 and Figure 2. The predicted probabilities of continuous characteristics were calculated at the 10th, 50th, and 90th percentiles of the sample distribution. Analyses were conducted using Stata statistical software (version 17, StataCorp). A 2-sided significance level of .05 was used.

## Results

### Sample Characteristics

The survey was sent to 1233 UAS panelists, of which 1135 completed the survey, a 92.1% cooperation rate. The demographics, health status, socioeconomic characteristics, financial literacy skills, and personality traits of the study sample are described in Table 1. Weighting for national representation, the sample included 652 women (51.2%) (95% CI, 47.0%-55.4%), 672 were married (59.6%) (95% CI, 55.3%-63.7%), 1051 (95% CI, 87.0%-92.7%) were born in the US (90.2%), and the age distribution was 103 (95% CI, 15.8%-23.8%) aged 20 to 34 years (19.5%), 366 (95% CI, 36.4%-44.8%) aged 35 to 54 years (40.5%), 260 (95% CI, 13.7%-19.2%) aged 55 to 64 years (16.3%), and 426 (95% CI, 20.8%-26.9%) aged 65 years or older (23.7%). The racial and ethnic composition was 89 (95% CI, 9.4%-15.5%) non-Hispanic Black (12.1%), 16.8% (95% CI, 13.3%-20.9%) Hispanic, 89 (95% CI, 56.8%-65.6%) non-Hispanic White (61.3%), and 88 (95% CI, 7.4%-13.2%) other non-Hispanic racial identity (9.9%). Whereas 57 (95% CI, 6.1%-11.3%) of those sampled were uninsured (8.3%), the remainder had private health insurance (682 [57.0%]; 95% CI, 52.8%-61.2%), Medicaid (140 [14.7%]; 95% CI, 11.8%-18.2%), Medicare (187 [11.9%]; 95% CI, 9.8%-14.4%), or Military coverage (27 [3.0%]; 95% CI, 1.8%-4.9%), and health insurance status was unknown for 42 (95% CI, 3.2%-7.6%) in the sample (5.0%). More than half (545 [54.4%]; 95% CI, 50.2%-58.5%) of respondents had no chronic conditions, though 276 (21.9%; 95% CI, 18.7%-25.5%) had 1, 147 (10.9%; 95% CI, 8.7%-13.6%) had 2, and 167 (12.8%; 95% CI, 10.4%-15.7%) had 3 or more. Income and education also vary in the study sample.

**Table 1.** Sample Characteristics

Characteristic	Total sample (N = 1135)		Has problematic bill (n = 203)		No problematic bill (n = 932)	
	No. (%)	95% CI, %	No. (%)	95% CI, %	No. (%)	95% CI, %
In the past 12 mo, have you or someone in your household ever received a medical bill that you did not agree with or could not afford to pay?						
Yes	203 (19.6)	16.4-23.1	203 (100)	NA	0	NA
No	932 (80.4)	76.9-83.6	0	NA	932 (100)	NA
Health insurance						
Private plan	682 (57.0)	52.8-61.2	120 (56.2)	46.4-65.5	562 (57.3)	52.5-61.9
Medicaid	140 (14.7)	11.8-18.2	28 (17.0)	10.4-26.5	112 (14.2)	11.1-18.0
Medicare	187 (11.9)	9.8-14.4	26 (7.2)	4.1-12.2	161 (13.1)	10.6-16.0
Military	27 (3.0)	1.8-4.9	3 (2.7)	0.8-9.1	24 (3.1)	1.8-5.3
Uninsured	57 (8.3)	6.1-11.3	19 (12.1)	6.9-20.2	38 (7.4)	5.0-10.8
Unknown	42 (5.0)	3.2-7.6	7 (4.9)	1.9-12.2	35 (5.0)	3.1-8.0
No. of chronic conditions						
None	545 (54.4)	50.2-58.5	74 (42.4)	33.2-52.2	471 (57.3)	52.6-61.8
1	276 (21.9)	18.7-25.5	54 (23.5)	16.7-32.1	222 (21.5)	18.0-25.5
2	147 (10.9)	8.7-13.6	29 (14.1)	8.8-21.9	118 (10.1)	7.8-13.0
≥3	167 (12.8)	10.4-15.7	46 (19.9)	13.1-29.0	121 (11.1)	8.7-14.0
Age, y						
20-34	103 (19.5)	15.8-23.8	14 (16.8)	9.7-27.4	89 (20.1)	16.0-25.0
35-54	366 (40.5)	36.4-44.8	85 (45.2)	35.8-54.8	281 (39.4)	34.8-44.2
55-64	240 (16.3)	13.7-19.2	55 (23.4)	16.6-31.9	185 (14.5)	11.9-17.5
≥65	426 (23.7)	20.8-26.9	49 (14.6)	9.7-21.4	377 (26.0)	22.6-29.6
Sex						
Female	652 (51.2)	47.0-55.4	133 (61.4)	51.8-70.1	519 (48.7)	44.0-53.5
Male	483 (48.8)	44.6-53.0	70 (38.6)	29.9-48.2	413 (51.3)	46.5-56.0
Married, yes	672 (59.6)	55.3-63.7	122 (64.9)	55.2-73.4	550 (58.3)	53.5-62.9
Race and ethnicity						
Non-Hispanic Black	89 (12.1)	9.4-15.5	19 (10.7)	6.0-18.3	70 (12.%)	9.3-16.4
Hispanic	103 (16.8)	13.3-20.9	19 (18.6)	11.8-28.3	84 (16.3)	12.5-21.0
Non-Hispanic White	855 (61.3)	56.8-65.6	145 (59.6)	49.5-68.9	710 (61.7)	56.6-66.5
Other^a^	88 (9.9)	7.4-13.2	20 (11.1)	5.8-20.3	68 (9.6)	6.9-13.3
Born in the US, yes	1051 (90.2)	87.0-92.7	193 (94.4)	87.4-97.6	858 (89.2)	85.4-92.1
Education						
No diploma	43 (7.7)	5.5-10.7	7 (6.5)	2.9-14.0	36 (8.0)	5.5-11.5
High school/some college	664 (56.6%)	52.4%-60.8%	124 (59.7%)	49.9-68.8	540 (55.9%)	51.1-60.6
Bachelor’s degree	233 (19.8)	16.5-23.5	36 (17.6)	11.3-26.6	197 (20.3)	16.7-24.6
Master’s/professional/doctorate degree	195 (15.9)	13.1-19.1	36 (16.2)	10.3-24.4	159 (15.8)	12.7-19.5
Household income, $^b^						
<25 000	192 (19.6)	16.4-23.4	35 (17.1)	10.6-26.4	157 (20.2)	16.6-24.4
25 000-49 999	257 (20.5)	17.4-23.9	52 (25.2)	18.0-34.1	205 (19.3)	16.1-23.0
50 000-74 999	201 (18.5)	15.4-22.2	33 (19.3)	12.6-28.3	168 (18.3)	14.9-22.4
75 000-149 999	324 (27.2)	23.6-31.1	56 (27.3)	19.7-36.6	269 (27.2)	23.2-31.6
≥150 000	160 (14.2)	11.4-17.5	27 (11.1)	6.5-18.5	133 (14.9)	11.8-18.7
	**No.**	**Median (10th, 90th percentiles)**	**No.**	**Median (10th, 90th percentiles)**	**No.**	**Median (10th, 90th percentiles)**
Financial literacy score (scale 0-14)	1135	10 (4-13)	203	9 (4-13)	932	10 (4-13)
Extroversion (scale 8-40)	1135	25 (17-34)	203	24 (15-34)	932	25 (17-34)
Agreeableness (scale 9-45)	1135	36 (27-42)	203	35 (27-42)	932	36 (27-43)
Conscientiousness (scale 9-45)	1135	36 (28-44)	203	35 (29-42)	932	36 (28-44)
Neuroticism (scale 8-40)	1135	21 (13-30)	203	23 (14-31)	932	21 (12-29)
Openness (scale 10-50)	1135	35 (28-43)	203	36 (27-44)	932	35 (28-43)

Abbreviation: NA, not applicable.

^a^ Other indicates participants who did not self identify as Black, Hispanic, or White.

^b^ Household income is missing for 1 respondent.

### Prevalence of Problematic Bills and Patient Response

Overall, 203 respondents (19.6%; 95% CI, 16.4%-23.1%) reported that their household received a bill that was unaffordable or they disagreed with (Table 1). Among those who received a problematic bill, 136 (61.5%; 95% CI, 51.6%-70.5%) reached out to the billing office and 67 (38.5%; 95% CI, 29.5%-48.4%) did not (Table 2).

**Table 2.** Prevalence of Problematic Bills and Patient Responses

Variable	No. (%) [95% CI]			
	Problematic bill reported (n = 203)	Among those reporting problematic bill		Comparing those who did and did not reach out, *P* value^a^
		Reached out (n = 136)	Did not reach out (n = 67)		
Thinking about the bill that was most concerning to you, did you or someone else reach out to the billing office?				NA^b^	
Yes	136 (61.5) [51.6-70.5]	136 (100.0)			
No	67 (38.5) [29.5-48.4]		67 (100.0)		
Thinking about the bill that was most concerning to you, what was the problem with the bill?					
Could not afford to pay the bill	81 (46.6) [37.1-56.4]	44 (39.2) [27.9-51.8]	37 (58.3) [41.8-73.1]	.06	
Felt the bill was unfairly high	88 (43.5) [34.3-53.2]	51 36.5 [25.9 48.5]	37 (54.6) [38.2 70.0]	.07	
Felt the bill was too high because of a mistake	68 (31.8) [23.6 41.4]	59 37.9 [27.3 49.9]	9 (22.2) [10.6 40.7]	.11	
The bill seemed confusing	54 (30.4) [22.2 40.1]	39 (34.5) [23.8 46.9]	15 (24.1) [12.8 40.7]	.29	
Thinking about the bill that was most concerning to you, what type of health care service was this for?				.13	
Physician office visit	66 (34.6) [25.8-44.5]	48 (34.5) [23.8-47.0]	18 (34.7) [20.6-52.1]		
Emergency department/urgent care center	33 (19.9) [13.3-28.8]	19 (18.0) [10.5-29.1]	14 (23.0) [11.9-39.7]		
Hospital	31 (15.3) [9.6-23.4]	17 (11.6) [5.9-21.5]	14 (21.1) [10.9-36.9]		
Radiograph, MRI, or scan	30 (13.4) [8.2-21.2]	23 (16.3) [9.3-26.9]	7 (8.7) [2.9-23.4]		
Laboratory	16 (6.1) [3.1-11.4]	11 (6.7) [3.0-14.3]	5 (5.0) [1.5-15.9]		
Outpatient procedure	10 (5.1) [2.2-11.6]	7 (7.5) [2.9-17.8]	3 (1.3) [0.3-5.1]		
Pharmacy	4 (1.9) [0.5-7.2]	0	4 (5.0) [1.2-18.0]		
Dental	5 (1.2) [0.4-3.5]	4 (1.5) [0.4-5.1]	1 (0.8) [0.1-5.7]		
Other	8 (2.6) [1.0-6.4]	7 (3.9) [1.5-10.2]	1 (0.4) [0.1-2.9]		
Thinking about when you or someone else reached out to the billing office, what was the reason?				NA^b^	
Ask questions about the price	NA^b^	77 (56.2) [44.0-67.8]	NA^b^		
Ask questions about the services	NA^b^	56 (47.2) [35.6-59.2]	NA^b^		
Ask about financial assistance	NA^b^	21 (19.0) [11.2-30.4]	NA^b^		
Set up a payment plan	NA^b^	26 (18.6) [11.3-29.2]	NA^b^		
Negotiate to pay less	NA^b^	20 (18.1) [10.4-29.5]	NA^b^		
Insurance issue	NA^b^	14 (4.2) [2.3-7.4]	NA^b^		
Other	NA	4 (5.0) [0.9-23.4]	NA		
What were the results of the communication with the billing office?					
Understand the bill better now	NA^b^	16 (18.2) [10.2-30.2]	NA^b^		
Bill was corrected	NA^b^	37 (25.7) [17.0-37.0]	NA^b^		
Set up a payment plan	NA^b^	18 (15.5) [8.7-26.3]	NA^b^		
Got financial assistance	NA^b^	10 (8.1) [3.5-17.9]	NA^b^		
Price was dropped	NA^b^	17 (15.2) [8.3-26.1]	NA^b^		
Bill was cancelled	NA^b^	6 (7.3) [2.1-22.6]	NA^b^		
Nothing changed	NA^b^	35 (23.9) [15.4-35.3]	NA^b^		
This problem is not solved yet	NA^b^	32 (21.8) [13.7-32.8]	NA^b^		
Why did anyone not reach out to the billing office about this bill?				NA^b^	
Did not think it would change the bill	NA^b^	NA^b^	55 (86.1) [72.3-93.7]		
Felt uncomfortable reaching out	NA^b^	NA^b^	18 (34.0) [20.2-51.1]		
Did not have time	NA^b^	NA^b^	12 (25.7) [13.4-43.6]		
Worried it would harm my medical care in the future	NA^b^	NA^b^	7 (8.7) [3.0-23.0]		
Did not know how	NA^b^	NA^b^	4 (3.7) [1.1-11.7]		
Other	NA^b^	NA^b^	7 (11.5) [4.6-26.0]		

Abbreviations: MRI, magnetic resonance imaging; NA, not applicable.

^a^ *P* values reported are results of χ^2^ tests comparing the attributes of respondents who did and did not reach out to the billing office, conditional on receiving a problematic bill.

^b^ Not applicable as the respondents were only asked certain questions conditional on reporting a problematic bill and/or reaching out to the billing office.

Respondents were asked to report the problem(s) with their bill among 4 options (select all that apply): 81 (46.6%; 95% CI, 37.1%-56.4%) could not afford to pay, 88 (43.5%; 95% CI, 34.3%-53.2%) felt the bill was unfairly high, 68 (31.8%; 95% CI, 23.6%-41.4%) felt the bill was too high because of a mistake, and 54 (30.4%; 95% CI, 22.2%-40.1%) reported that the bill seemed confusing (Table 2). The sources of the bills were physician offices (66 [34.6%]; 95% CI, 25.8%-44.5%), emergency room or urgent care (33 [19.9%]; 95% CI, 13.3%-28.8%]), hospital (31 [15.3%]; 95% CI, 9.6%-23.4%), imaging (30 [13.4%]; 95% CI, 8.2%-21.2%), laboratory (16 [6.1%]; 95% CI, 3.1%-11.4%), outpatient procedures (10 [5.1%]; 95% CI, 2.2%-11.6%), pharmacy (4 [4%]), dental (5 [5%]), and other (8 [2.6%]; 95% CI, 1.0%-6.4%). Using χ^2^ tests, there was not a statistically significant difference between those who did and did not reach out to the billing office with respect to the prevalence of each of these problems nor source of the bill (Table 2).

Those who reported that nobody reached out to the billing office about the problematic bill were asked to select reasons why from a list (select all that apply) (Table 2). Fifty-five (86.1%; 95% CI, 72.3%-93.7%) reported that they did not think it would change the bill. Eighteen (34.0%; 95% CI, 20.2%-51.1%) did not feel comfortable reaching out, and one-quarter (25.7%; 95% CI, 13.4%-43.6%) did not have time. Overall, 7 (8.7%; 95% CI, 3.0%-23.0%) respondents worried reaching out would harm future care, and 4 (3.7%; 95% CI, 1.1%-11.7%) did not know how to reach out.

Those who reported that someone did reach out to the billing office were asked to select objectives from a list (select all that apply) (Table 2). Many wanted to ask questions about price (77 [56.2%]; 95% CI, 44.0%-67.8%) and/or services (56 [47.2%]; 95% CI, 35.6%-59.2%). About 1 in 5 were seeking financial assistance (21 [19.0%]; 95% CI, 11.2%-30.4%), a payment plan (26 [18.6%]; 95% CI, 11.3%-29.2%), and/or negotiating to pay less (20 [18.1%]; 95% CI, 10.4%-29.5%). Four percent (14 [4.2%]; 95% CI, 2.3%-7.4%) reached out with an insurance issue.

### Characteristics of Individuals Receiving Problematic Bills and Reaching Out

Having more chronic conditions and being female was associated with increased likelihood of reporting receiving a problematic bill, after controlling for insurance type, age, education, income, marital status, and race and ethnicity (Figure 1A). Respondents with 3 or more chronic conditions had a 32.4% (95% CI, 21.0%-43.9%) predicted probability of receiving a problematic bill, compared with 12.8% (95% CI, 9.0%-16.6%) for those with no chronic conditions. Female and male participants had a 22.0% (95% CI, 17.1%-27.0%) and 13.6% (95% CI, 9.5%-17.7%) predicted probability of receiving a problematic bill, respectively. In a separate model, higher neuroticism was associated with greater likelihood of reporting receiving a problematic bill, after controlling for financial literacy and other personality traits (Figure 1B).

Conditional on receiving a bill, respondents who were uninsured or Medicare beneficiaries, less educated, and in the 35 to 44 year age range were less likely to reach out to the billing office, whereas those with military insurance were more likely to reach out, after controlling for other demographic, socioeconomic, and health attributes (Figure 1C). The predicted probability of reaching out was 19.9% (95% CI, −6.7% to 46.6%) among those without a high school diploma, and above 60% for all higher education groups. Respondents with private insurance had a 68.1% (95% CI, 54.3%-82.0%) predicted probability of reaching out compared with 32.5% (95% CI, 4.2%-60.7%) among the uninsured and 30.1% (95% CI, 1.0%-59.3%) among Medicare beneficiaries. Higher financial literacy, higher extroversion, and lower agreeableness were also associated with greater likelihood of reaching out, after controlling for other personality traits (Figure 1D).

### Outcomes of Reaching Out to Billing Offices

For 32 (95% CI, 13.7%-32.8%) respondents (21.8%) who reported that their household reached out about a household bill, their problem was not solved yet (eTable 1 in Supplement 1). Among the remaining respondents, nothing changed for 30.6% (95% CI, 19.7%-44.2%) of them. One-third (32.9%; 95% CI, 21.8%-46.4%) had a bill corrected and 23.2% (95% CI, 13.2%-37.6%) reported a better understanding of their bill. Many received financial relief in the forms of bill cancellation (9.3%; 95% CI, 2.7%-27.9%), financial assistance (10.4%; 95% CI, 4.4%-22.5%), price reduction (19.4%; 95% CI, 10.7%-32.7%), and/or setting up a payment plan (19.8%; 95% CI, 11.1%-32.9%). These outcomes are not mutually exclusive; many respondents reported multiple outcomes.

We evaluated resolutions stratified by the bill problem and reasons for reaching out (eTable 2 in Supplement 1). Among 34 participants who could not afford their bill and reached out, 14 (49.0%; 95% CI, 26.3%-72.0%) received financial aid, bill cancellation, and/or price reductions. An additional 11 (26.8%; 95% CI, 11.5%-50.8%) received a payment plan. Overall, 75.8% of the respondents who could not afford their bill and reached out received some form of financial relief. Among 37 participants who thought their bill was too high because of a mistake and reached out, 25 (73.7%; 95% CI, 50.2%-88.7%) had their bill corrected. Among 14 participants who reached out wanting to negotiate the price, 10 (61.8%; 95% CI, 10.6%-76.3%) reported that the price was reduced.

### Process and Experience of Reaching Out to Billing Offices

Overall, 104 survey respondents communicated with the billing office personally (69.2%; 95% CI, 57.1%-79.1%), whereas 35 reported another household member (30.7%; 95% CI, 20.9%-42.5%) or someone from an advocacy organization (4 [3.5%]; 95% CI, 1.0%-11.6%) reaching out (Table 3). Among those who reached out personally, 96 did so by telephone (87.4%; 95% CI, 74.1%-94.4%) and spent less than 1 hour reaching out (66 [60.2%]; 95% CI, 45.6%-73.2%). The majority strongly agreed (49 [45.7%]; 95% CI, 32.1%-60.0%) or agreed (39 [47.3%]; 95% CI, 33.3%-61.8%) that they were comfortable communicating with the billing office. Whereas 28 (23.4%; 95% CI, 14.0%-36.5%) strongly agreed and 33 (31.2%; 95% CI, 19.0%-46.8%) agreed that they felt treated with respect by the people in the billing office, 10 (11.0%; 95% CI, 4.6%-23.9%) disagreed, and 9 (7.8%; 95% CI, 3.3%-17.6%) strongly disagreed.

**Table 3.** Process and Experience of Reaching Out to Billing Offices

Variable	No. (%) [95% CI]
**Among those reporting a problematic bill who reached out (n = 136)**	
Who communicated with the billing office?	
Me	104 (69.2) [57.1-79.1]
Another household member	35 (30.7) [20.9-42.5]
Somebody from an advocacy organization	4 (3.5) [1.0-11.6]
A friend or relative	0
**Among those who reached out personally (n = 104)**	
How did you communicate with the billing office?	
Telephone	96 (87.4) [74.1-94.4]
Letters	8 (9.8) [3.9-22.4]
Emails	14 (12.0) [5.9-23.0]
In person	9 (16.2) [7.8-30.8]
What was the total amount of time you spent communicating with the billing office?	
<1 h	66 (60.2) [45.6-73.2]
1-2 h	23 (22.9) [13.1-36.8]
2-5 h	10 (11.9) [5.1-25.2]
>5 h	5 (5.0) [1.3-17.3]
Overall, I felt comfortable communicating with the billing office myself.	
Strongly agree	49 (45.7) [32.1-60.0]
Agree	39 (47.3) [33.3-61.8]
Neutral	10 (5.3) [2.6-10.3]
Disagree	5 (1.3) [0.4-3.8]
Strongly disagree	1 (0.4) [0.1-2.9]
Overall, I felt the people working at the billing office treated me with respect.	
Strongly agree	28 (23.4) [14.0-36.5]
Agree	33 (31.2) [19.0-46.8]
Neutral	24 (26.5) [15.7-41.2]
Disagree	10 (11.0) [4.6-23.9]
Strongly disagree	9 (7.8) [3.3-17.6]

## Discussion

In this cross-sectional survey of a representative sample of people in the US, 1 in 5 reported that their household received a medical bill that they could not afford or disagree with and more than half of those (61.5%) reported that someone reached out to the billing office to address the concern. Whereas most respondents (86.1%) who did not reach out reported that they did not think it would make a difference, the experiences of those who did reach out provide evidence to the contrary. Most respondents who reached out reported at least 1 form of financial relief, bill corrections, or better understanding of the bill.

These findings suggest that patients benefit from reaching out to the billing office on receiving a problematic bill, and those who do not reach out may be missing financially advantageous opportunities. The likelihood of receiving problematic bills did not significantly differ by insurance type, education, or income. However, conditional on receiving a problematic bill, those who were uninsured, Medicare insured, and less educated were less likely to reach out to the billing office. These differences in self-advocacy may be widening the gap in financial burden of health care between those with higher and lower socioeconomic status. This is consistent with literature that found administrative burden yields disparate impacts across populations.^15^

This study also demonstrates the role of personality traits in self-advocacy behaviors, whereby a more extroverted and less agreeable personality was associated with increased likelihood of reaching out. Only 3.5% of respondents had help from an advocacy organization to reach out to the billing office, and the remaining cases where handled directly by households. Among those who reached out to the billing office themselves, the time burden stretched from an hour or less (60.2% of people) to more than 5 hours (5.0% of people). Nearly 1 in 5 (18.8%) respondents who reached out personally did not feel respected by the billing office staff. These process burden findings, paired with the finding that 38.5% of respondents did not reach out at all, suggests that there may be unmet need for formal assistance navigating bills.

Nearly one-third (31.8%) of respondents with a problematic bill suspected that their bill was too high because of a billing error. Among those who reached out about a billing error, most (73.7%) reported that a mistake on the bill was corrected. This indicates that billing errors are not uncommon and self-advocacy to correct errors is often successful. Consumer advocates report that common billing errors include double billing and upcoding, inaccuracies when medical billers create claims without complete clinical information, and erroneous reductions or denials on the part of insurers adjudicating claims.^16^

Efforts to streamline the administrative burden of addressing billing issues may improve access to financial relief for more patients. For example, as of 2021 there were 13 states that mandated hospitals screen patients for insurance eligibility and eligibility for other programs, including the entities’ charity or discounting policy.^17^ Policies that shift administrative responsibility from patients to the billing health care professional may counter the self-advocacy disparities observed in this study.

### Limitations

A study limitation is the relatively small survey sample, though the cooperation rate was high at 92.1% and the weighting schema was designed to yield national representation. This was a novel survey exploring patient self-advocacy experience and outcomes, and future studies could build on this one with an expanded scope of questions and larger sample size. Respondent sociodemographic characteristics, financial literacy, and personality traits were captured on a prior survey rather than concurrently with this survey. The study also captured reported behavior from the prior 12 months, which may be subject to recall bias and variation in follow-up time since bill receipt.

## Conclusions

This cross-sectional survey study found that 40% of households that received a bill they could not afford or disagreed with did not contact the billing office. Most of those who did self-advocate achieved bill corrections and payment relief. Respondents with lower education levels, those with less financial literacy, and the uninsured were less willing or able to reach out to a billing office regarding their problematic bill, and therefore likely missed opportunities to alleviate their bills. These differences in self-advocacy may be exacerbating socioeconomic inequalities in medical debt burden.

## Notes

Supplement 1. eAppendix. eTable 1. Outcomes of Reaching Out to Billing Offices eTable 2. Outcomes by Billing Concern eTable 3. Regression Results

Supplement 2. Data Sharing Statement
