# Supplementary material for: Disparate Patient Advocacy When Facing Unaffordable and Problematic Medical Bills
Source: JAMA Health Forum. 2024 Aug 30;5(8):e242744. doi: 10.1001/jamahealthforum.2024.2744 (PMC11364993; doi:10.1001/jamahealthforum.2024.2744)
Supplement: Supplement 1. — eAppendix. eTable 1. Outcomes of Reaching Out to Billing Offices eTable 2. Outcomes by Billing Concern eTable 3. Regression Results [file jamahealthforum-e242744-s001.pdf]

Supplemental Online Content

Duffy EL, Frasco MA, Trish E. Disparate patient advocacy when facing unaffordable and problematic medical bills. *JAMA Health Forum*. 2024;5(8):e242744.
doi:10.1001/jamahealthforum.2024.2744

eAppendix.

eTable 1. Outcomes of Reaching Out to Billing Offices

eTable 2. Outcomes by Billing Concern

eTable 3. Regression Results

This supplemental material has been provided by the authors to give readers additional information about their work.

© 2024 Duffy EL et al. *JAMA Health Forum.*

eAppendix

Understanding America Study internet panel study design

The Understanding America Study is a probability-based internet panel of American households maintained by the Center for Economic and Social Research at the University of Southern California.[1] The initial panel was drawn using address-based sampling in zip codes that were selected based on certain sociodemographic dimensions. Prospective respondents were recruited by mailing a pre-notification letter followed by a paper survey. On the last page of the survey, respondents were asked if they wanted to join the panel to regularly participate in online surveys using a computer, tablet, or smartphone. The response rate to the initial survey was 41.3%.[2] The panel consists of approximately 13,000 individuals ages 18 and older and is representative of the US general population with respect to gender, race-ethnicity, age, education, and census region.

We designed and fielded a survey to a sample of 1,233 panelists who were aged 20 and older. The cooperation rate for our survey was 92.0%. Survey-specific sample weights were generated using two steps: weights to correct for unequal probabilities of sampling for the initial survey and a final post-stratification weight allowing the survey data to be representative of the US population.

Survey Instrument

Instructions: In this section, you will be asked questions about recent **medical bills** you may have received.

Question 10: In the **past 12 months**, have you or someone in your household ever received a medical bill that you didn't agree with or couldn't afford to pay?

1. Yes
2. No

[NOTE to programmers: Please show next questions if respondent answered 'Yes' in Question 10]

Q10YESa: Thinking about the bill that was most concerning to you, **what was the problem with the bill**?

[Check all that apply]

1. Could not afford to pay the bill
2. Felt the bill was unfairly high
3. Felt the bill was too high because of a mistake
4. The bill seemed confusing

---

[1] Alattar L, Messel M, Rogofsky D. An introduction to the Understanding America Study internet panel. *Soc Sec Bull*. 2018;78:13.
[2] Understanding America Study: Response and Attrition. Accessed April 19, 2024. https://uasdata.usc.edu/index.php

Q10YESb: Thinking about the bill that was most concerning to you, **what type of health care service was this for**?

1. Hospital
2. Doctor office visit
3. Emergency Room (ER) or urgent care center
4. Pharmacy
5. X-ray, MRI, or scan
6. Laboratory
7. Other; describe: _[free response]________

Q10YESc: Thinking about the bill that was most concerning to you, **did you or someone else reach out to the billing office**?

1. Yes
2. No

[NOTE to programmers: Please show next question if respondent answered 'No' in **Q10YESc**]

Q10YEScNO: Why didn't anyone reach out to the billing office about this bill?

[select all that apply]

1. Felt uncomfortable reaching out
2. Didn't have time
3. Didn't know how
4. Didn't think it would change the bill
5. Worried it would harm my medical care in the future
6. Other; describe: [free text]

[NOTE to programmers: Please show following questions if respondent answered 'YES' in **Q10YESc**]

Q10YESc1: Who communicated with the billing office?

[check all that apply]

1. Me
2. Another household member
3. A friend or relative
4. Somebody from an advocacy organization (for example, Dollar For)

[NOTE to programmers: Please show following questions if respondent answered '1. ME' in **Q10YESc1**]

© 2024 Duffy EL et al. *JAMA Health Forum.*

[NOTE to programmers: Please skip to **Q10YESc2** if respondent did not answer ‘1. ME’ in **Q10YESc1**]

Q10YESc1MEa. Overall, I felt comfortable communicating with the billing office myself.

1. Strongly agree
2. Agree
3. Neutral
4. Disagree
5. Strongly disagree

Q10YESc1MEb. Overall, I felt the people working at the billing office treated me with respect.

1. Strongly agree
2. Agree
3. Neutral
4. Disagree
5. Strongly disagree

Q10YESc1MEc: How did you communicate with the billing office?

[check all that apply]

1. Telephone
2. Letters
3. Emails
4. Other; describe: [free text]

Q10YESc1MEd: What was the total amount of time you spent communicating with the billing office?

1. Less than 1 hour
2. 1-2 Hours
3. 2-5 Hours
4. More than 5 hours

Q10YESc2: Thinking about when you or someone else reached out to the billing office, what was the reason?

[check all that apply]

1. Ask questions about the services
2. Ask questions about the price
3. Ask about financial assistance
4. Negotiate to pay less

© 2024 Duffy EL et al. *JAMA Health Forum.*

5. Set up a payment plan
6. Other; describe: [free text]

Q10YESc3: What were the results of the communication with the billing office?

[check all that apply]

1. Understand the bill better now
2. Bill was corrected
3. Set up a payment plan
4. Got financial assistance
5. Price was dropped
6. Bill was cancelled
7. Nothing changed
8. This problem is not solved yet

© 2024 Duffy EL et al. *JAMA Health Forum.*

Measures

Sociodemographic characteristics are reported in Table 1 and employed in regression analysis using the following forms:

Age: Categorical variable with categories for 20-34, 35-54, 55-64, and ≥65 years old.

Gender: Binary (male, female) categorical variable.

Marital status: Binary (married, not married) categorical variable.

Race/ethnicity: Categorical variable with categories for White non-Hispanic, Black non-Hispanic, Hispanic, and Other. Respondents who reported multiple races were categorized with priority to their status as Hispanic, Black, and White.

National origin: Binary (born in the US, not born in the US) categorical variable.

Education: Categorical variable identifying the highest level of education as No diploma, High School/Some college, Bachelor, or Masters/Professional/Doctorate.

Household income: Categorical variable with levels for less than $25,000; $25,000-$49,999; $50,000-$74,999; $75,000-$149,999; and $150,000 or more.

Insurance type: Categorical variable identifying uninsured and private, Medicaid, Medicare, and military coverage types. There is also an unknown insurance status category. Respondents with multiple insurer types were assigned to one using the following hierarchy: private, military, Medicaid, Medicare, uninsured, and unknown.

Number of chronic conditions: Categorized as having none, one, two, and three or more of the following conditions: asthma, emphysema, bronchitis, arthritis, cancer, diabetes, digestive problems, heart trouble, immunocompromised condition, kidney disease, liver problem, and stroke.

© 2024 Duffy EL et al. *JAMA Health Forum.*

eTable 1. Outcomes of Reaching Out to Billing Offices

	Among Those Reporting Problematic Bill					
	**Reached Out N=136**			**Reached Out & Resolved N=104**		
What were the results of the communication with the billing office?	N	**Percent**	[95% conf. interval]	N	**Percent**	[95% conf. interval]
Understand the bill better now	16	**18.2%**	[10.2% - 30.2%]	16	**23.2%**	[13.2% - 37.6%]
Bill was corrected	37	**25.7%**	[17.0% - 37.0%]	37	**32.9%**	[21.8% - 46.4%]
Set up a payment plan	18	**15.5%**	[8.7% - 26.3%]	18	**19.8%**	[11.1% - 32.9%]
Got financial assistance	10	**8.1%**	[3.5% - 17.9%]	10	**10.4%**	[4.4% - 22.5%]
Price was dropped	17	**15.2%**	[8.3% - 26.1%]	17	**19.4%**	[10.7% - 32.7%]
Bill was cancelled	6	**7.3%**	[2.1% - 22.6%]	6	**9.3%**	[2.7% - 27.9%]
Nothing changed	35	**23.9%**	[15.4% - 35.3%]	35	**30.6%**	[19.7% - 44.2%]
This problem is not solved yet	32	**21.8%**	[13.7% - 32.8%]	0		

Note: Responses are not mutually exclusive. The observations in the “Reached Out & Resolved” column are a subset of those in the “Reached Out” column.

© 2024 Duffy EL et al. *JAMA Health Forum.*

eTable 2. Outcomes by Billing Concern

	N	Among Those Reporting Problematic Bill, Reached Out & Resolved<br>Percent	[95% conf. interval]	
**Bill Problem: Could Not Afford (N=35)**				
Change in amount owed (got financial aid, bill cancelled, and/or price dropped)	14	49.0%	[26.3%,	72.0%]
No change in amount owed, but set up payment plan	11	26.8%	[11.5%,	50.8%]
No financial change	10	24.2%	[10.7%,	46.0%]
**Bill Problem: Mistake (N=37)**				
Mistake corrected	25	73.7%	[50.2%,	88.7%]
No mistake corrected	12	26.3%	[11.3%,	49.8%]
**Reached Out Seeking to Negotiate Price (N=14)**				
Got price drop	10	61.8%	[10.6%,	76.3%]
No price drop	4	38.2%	[23.7%,	89.4%]

Note: This table describes resolutions among respondents who reached out with select concerns about affordability, mistakes, or a desire to negotiate price. Not all respondents who received a problematic bill and reached out are included in this table.

© 2024 Duffy EL et al. *JAMA Health Forum.*

eTable 3. Regression Results

	(1) Problematic Bill	(2) Problematic Bill	(3) Reached Out	(4) Reached out
Coverage (ref=Private): Medicaid	0.0334		0.152	
Medicare	-0.0156		-0.223	
Military	-0.0123		0.240**	
Uninsured	0.111		-0.287**	
Unknown	0.0305		-0.114	
Age (ref=20-34): 35-44	0.0542		-0.332**	
55-64	0.0873		-0.0276	
65 and over	-0.0695		0.223	
# Chronic Conditions (ref=0): 1	0.0657		-0.169*	
2	0.144**		-0.130	
3 or more	0.185***		-0.105	
Education (ref=no degree): High School/Some college	0.0417		0.292**	
Bachelor	0.0636		0.296*	
Masters/Professional/Doctorate	0.0810		0.417**	
Household income (ref=<$25,000): $25,000-$49,999	0.0853		0.112	
$50,000-$74,999	0.0649		-0.0397	
$75,000-$149,999	0.0342		0.245*	
$150,000 or more	-0.0259		0.214	
Race & Ethnicity (ref=White): Black non-Hispanic	-0.00931		0.172	
Hispanic	0.0106		0.0889	
Other	0.0253		-0.0148	
Male (ref=female)	-0.0780**		0.0777	
Married (ref=not married)	0.0586		-0.100	
Financial Literacy Score (maximum of 14)		0.00234		0.0356***
Extroversion Score (maximum of 40)		-0.000776		0.0194***
Agreeableness Score (maximum of 45)		0.000256		-0.0175**
Conscientiousness Score (maximum of 45)		0.00208		0.00450
Neuroticism Score (maximum of 40)		0.0114***		-0.0141*
Openness Score (maximum of 50)		0.00437		-0.00513
Constant	0.0237	-0.286	0.436*	0.800
Observations	1,134	1,135	202	203
R-squared	0.070	0.031	0.349	0.198

Standard errors in parentheses
*** p<0.01, ** p<0.05, * p<0.1

Note: Household income is missing for one respondent. This table reports on four multivariable probit regression models. Model 1 assesses the association between respondents' sociodemographic characteristics and the outcome of having received a problematic bill. Model 2 assesses the association between respondents' financial literacy and personality traits and the outcomes of having received a problematic bill. Inclusion in Models 3 and 4 is conditional on having received a problematic bill. Model 3 assesses the association between respondents' sociodemographic characteristics and

© 2024 Duffy EL et al. *JAMA Health Forum.*

the outcome of reaching out to the billing office. Model 4 assesses the association between respondents’ financial literacy and personality traits and the outcome of reaching out to the billing office. Results of these probit regression models were used to derive the predicted probabilities reported in Figures 1 and 2. Model 1 corresponds to Figure 1 Panel A and Model 2 corresponds to Figure 1 Panel B. Models 3 and 4 correspond to Figure 2 Panels A and B, respectively.

© 2024 Duffy EL et al. *JAMA Health Forum.*
