# Supplementary material for: Disparate Patient Advocacy When Facing Unaffordable and Problematic Medical Bills
Source: JAMA Health Forum. 2024 Aug 30;5(8):e242744. doi: 10.1001/jamahealthforum.2024.2744 (PMC11364993; doi:10.1001/jamahealthforum.2024.2744)
Supplement: Supplement 2. — Data Sharing Statement [file jamahealthforum-e242744-s002.pdf]

Data Sharing Statement

Duffy. Disparate Patient Advocacy When Facing Unaffordable and Problematic Medical Bills. *JAMA Health Forum*. Published August 30, 2024. doi:10.1001/jamahealthforum.2024.2744

Data
Data available: Yes
Data types: Deidentified participant data
How to access data: De-identified data for this survey study and a data dictionary will be made available on the Understanding America Study website (https://uasdata.usc.edu/index.php). Parties must complete a Data Use Agreement to access the data.
When available: With publication

Supporting Documents
Document types: None

Additional Information
Who can access the data: Anyone requesting the data.
Types of analyses: For any purpose.
Mechanisms of data availability: Parties must complete a Data Use Agreement to access the data.
